# Genetic and functional analyses of *SPTLC1* in juvenile amyotrophic lateral sclerosis

**DOI:** 10.1007/s00415-024-12776-5

**Published:** 2024-12-12

**Authors:** So Okubo, Hiroya Naruse, Hiroyuki Ishiura, Atsushi Sudo, Kayoko Esaki, Jun Mitsui, Takashi Matsukawa, Wataru Satake, Peter Greimel, Nanoka Shingai, Yasushi Oya, Takeo Yoshikawa, Shoji Tsuji, Tatsushi Toda

**Affiliations:** 1 Department of Neurology, Graduate School of Medicine, The University of Tokyo, 7-3-1 Hongo, Bunkyo-ku, Tokyo, 113-8655 Japan; 2 Department of Precision Medicine Neurology, Graduate School of Medicine, The University of Tokyo, Tokyo, Japan; 3 Department of Neurology, Okayama University Graduate School of Medicine, Dentistry and Pharmaceutical Sciences, Okayama, Japan; 4 Department of Biotechnology and Life Sciences, Faculty of Biotechnology and Life Sciences, Sojo University, Kumamoto, Japan; 5 Laboratory for Cell Function Dynamics, RIKEN Centre for Brain Sciences, Wako, Saitama Japan; 6 Division of Applied Life Science, Graduate School of Engineering, Sojo University, Kumamoto, Japan; 7 Department of Neurology, National Center of Neurology and Psychiatry, Tokyo, Japan; 8 Laboratory of Molecular Psychiatry, RIKEN Center for Brain Science, Wako, Saitama Japan; 9 Institute of Medical Genomics, International University of Health and Welfare, Chiba, Japan

**Keywords:** Juvenile amyotrophic lateral sclerosis, *SPTLC1*, Sphingolipids, Mosaicism

## Abstract

**Introduction:**

Amyotrophic lateral sclerosis (ALS) is a progressive neurodegenerative disorder of the motor system. Pathogenic variants in *SPTLC1*, encoding a subunit of serine palmitoyltransferase, cause hereditary sensory and autonomic neuropathy type 1 (HSAN1), and have recently been associated with juvenile ALS. *SPTLC1* variants associated with ALS cause elevated levels of sphinganines and ceramides. Reports on ALS associated with *SPTLC1* remain limited. This study aimed to investigate the frequency of *SPTLC1* variants in ALS and relevant clinical characteristics.

**Methods:**

We analyzed whole-exome and whole-genome sequence data from 40 probands with familial ALS and 413 patients with sporadic ALS without previously identified causative variants. Reverse transcription polymerase chain reaction (RT-PCR) analysis and droplet digital PCR (ddPCR) were used to assess splicing and mosaicism, respectively. Plasma sphingolipid levels were quantified to analyze biochemical consequences.

**Results:**

The heterozygous c.58G>A, p.Ala20Thr variant was identified in a 21-year-old Japanese female patient presenting with symmetric weakness which slowly progressed over 15 years. RT-PCR analysis showed no splice defects. Plasma sphingolipid levels in the patient were significantly increased compared to her asymptomatic parents. ddPCR revealed that the asymptomatic father harbored a mosaic variant with 17% relative mutant allele abundance in peripheral blood leukocytes.

**Conclusions:**

We identified a pathogenic c.58G>A, p.Ala20Thr *SPTLC1* variant in a patient with juvenile ALS, likely inherited from an asymptomatic parent with mosaicism. Lipid analysis results are consistent with previous findings on *SPTLC1*-associated ALS. Further studies are necessary to determine the clinical effect of mosaic variants of *SPTLC1*.

**Supplementary Information:**

The online version contains supplementary material available at 10.1007/s00415-024-12776-5.

## Introduction

Amyotrophic lateral sclerosis (ALS) is a neurological disorder characterized by progressive neurodegeneration of the motor system. Most ALS cases are sporadic; familial cases account for only 5–10% of ALS cases [1–3]. We have previously reported that causative genetic variants are identified in 60.3% of familial cases [4]. Most patients with ALS have an onset of disease in adulthood. Juvenile ALS is a rare form of ALS typically defined as an onset earlier than 25 years. Juvenile ALS is more frequently genetic in origin than adult-onset ALS [5]. Pathogenic variants in *ALS2*, *FUS*, and *SPG11* are among the most commonly found in patients with juvenile ALS. Several other genes have also been reported to be associated with juvenile ALS, including *SETX*, *SOD1*, *UBQLN2*, and *SIGMAR1* [6, 7]*.*

Serine palmitoyltransferase long chain base subunit 1 (*SPTLC1*) encodes a subunit of serine palmitoyltransferase (SPT). Pathogenic variants in *SPTLC1* are associated with hereditary sensory and autonomic neuropathy type 1 (HSAN1) [8]. More recently, other *SPTLC1* variants cause juvenile ALS [9, 10]. These variants cluster in exon 2 encoding the first transmembrane domain of SPTLC1. Notably, the biochemical consequences of variants associated with ALS result in elevated levels of sphinganines (SAs) and ceramides because of the unregulated excessive activity of SPT due to the loss of inhibition by ORMDLs. Meanwhile, SPTLC1, with variants associated with HSAN1, preferentially uses l-alanine instead of l-serine as a substrate, resulting in the aberrant synthesis of deoxysphingolipids. Similarly, elevated levels of SAs and ceramides have been observed in recently reported ALS cases associated with pathogenic variants in *SPTLC2*, another gene encoding a subunit of SPT [11, 12].

In this study, we identified a pathogenic *SPTLC1* variant in a patient with juvenile ALS. Lipid analysis revealed elevated levels of SA and ceramide, consistent with previous reports of *SPTLC1*-associated ALS. Interestingly, the patient’s father had mosaicism for the pathogenic *SPTLC1* variant, but was neurologically intact. We aimed to interpret this phenomenon by comparing the sphingolipid levels in three family members with different mutant allele burdens.

## Methods

### Participants and genetic analysis

In our recent study of 62 ALS probands with an onset age < 40 years, we identified two pathogenic *SPTLC2* variants: one in a family with familial ALS (FALS) and one in a patient with sporadic ALS (SALS) [12]. In this study, to avoid overlaps with our previous report, we aimed to investigate the frequency of *SPTLC1* variants through reanalysis of whole-exome or whole-genome sequence data of 453 patients with ALS who have not been analyzed for variants in *SPTLC1* in our previous reports, including 412 patients with SALS with age at onset ≥ 40 years, one patient with SALS with age at onset < 40 years, and 40 patients with FALS with age at onset ≥ 40 years. All patients were diagnosed with clinically definite, probable, laboratory-supported probable, or possible ALS according to the revised El Escorial criteria [13]. In addition, 1163 unrelated healthy individuals without a reported history of ALS or FTLD were included as control DNAs.

We analyzed whole-exome and whole-genome sequence data, as described previously [4, 12, 14–16] and in the Supplemental Methods. We extracted rare variants, including missense, nonsense, splice-site, and indels, from the *SPTLC1* gene. To focus on rare variants, variants with a minor allele frequency (MAF) ≥ 0.01 in any of the following population databases were excluded from the analysis: the East-Asian gnomAD (The Genome Aggregation Database; https://gnomad.broadinstitute.org/) and ToMMo 54KJPN-SNV/INDEL Allele Frequency Panel (https://jmorp.megabank.tohoku.ac.jp), all of which were last accessed in October 2023. Genomic DNA was extracted from peripheral blood leukocytes using standard procedures. Variants were confirmed by direct nucleotide sequence analysis using an ABI 3730 Genetic Analyzer. Functional prediction of variants was conducted using the Combined Annotation Dependent Depletion [17], Mendelian Clinically Applicable Pathogenicity score [18], and Functional, Molecular and Phenotypic Consequences of Amino Acid Substitutions Using Hidden Markov Models [19].

### Complementary DNA analysis of mutant *SPTLC1*

**Fig. 1:**
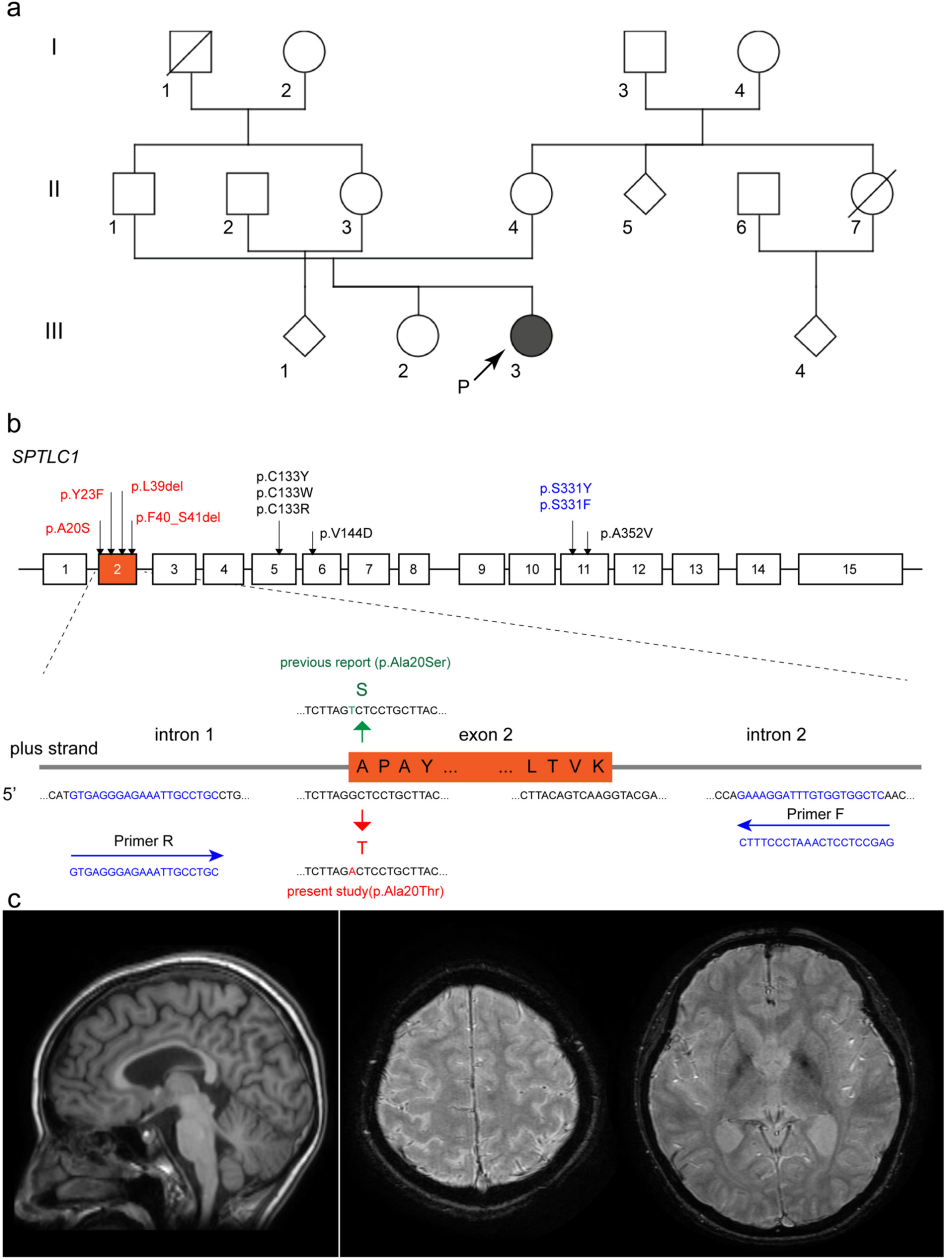
**a** Pedigree chart of a patient with early-onset amyotrophic lateral sclerosis (ALS) carrying the serine palmitoyltransferase long chain base subunit 1 (*SPTLC1*) variant. Arrows indicate the proband. Filled and open symbols represent ALS-affected and ALS-unaffected individuals, respectively, and slashed symbols indicate deceased individuals. Squares represent males, and circles denote females. **b** Physical map of SPTLC1 and primers used for polymerase chain reaction analysis. Variants associated with juvenile ALS and HSAN1 are shown in red and black, respectively. Variants in the S331 residue, indicated in blue, demonstrate an overlapping phenotype of HSAN and motor neuron disease. **c** Magnetic resonance imaging (MRI) studies of the brain. Sagittal view of T1-weighted imaging (left), and axial view of susceptibility weighted imaging (middle, right) are shown

To evaluate the effect of variants in *SPTLC1* on pedigree 1 mRNA expression (Fig. 1a), we performed reverse transcription polymerase chain reaction (RT-PCR) analysis. Total RNA was extracted from cultured lymphoblastoid cells established from the proband (III-3) with *SPTLC1* (NM_006415.4) c.58G>A variant. RNA was reverse-transcribed into complementary DNA (cDNA) using ReverTra Ace^®^ qPCR RT Master Mix (containing random and oligo dT primers) with gDNA Remover (Qiagen) following the manufacturerʼs protocol. The resulting cDNA was subsequently used for PCR amplification using the gene-specific primers shown (Supplemental Table 1), followed by electrophoresis through a 1% agarose gel. A direct nucleotide sequence analysis was performed to confirm the sequences of the cDNA products.

### Droplet digital polymerase chain reaction analysis

To assess mosaicism in pedigree 1, we determined the copy number of mutant *SPTLC1* in the peripheral blood leukocytes of the proband (III-3) and her parents (II-1, II-4) using QX200 droplet digital PCR (ddPCR) (Bio-Rad, CA) using two wells for each sample. Twenty nanograms of genomic DNA prepared from the peripheral blood leukocytes of the patient and her parents was mixed with ddPCR Supermix for probes (no dUTP), Mse I (5 units/well), the target probe for wildtype *SPTLC1* labeled with 5′ HEX, target probe for mutant *SPTLC1* labeled with 5′ 6-FAM, forward primer (5′-TCCAGAGGATCAGAATCC-3′), and reverse primer (5′-GGTGTTAGAAGTGTATGTTTC-3′). Droplet generation, PCR, and droplet analysis were performed according to manufacturer’s instructions. The sequences of the probes and primers used for the analysis are summarized in Supplemental Table 1.

### Sphingolipid measurements

Lipid analysis was performed on plasma samples from pedigree 1. Sphingolipids were extracted from the samples with chloroform/methanol (1:2, v/v) and quantified using liquid chromatography–electrospray ionization–tandem mass spectrometry [20]. The details of which are described in Supplemental Methods. Each sample underwent three to four independent measurements.

### Statistical analyses

All statistical evaluations of the sphingolipid analyses were conducted using Microsoft Excel and R Statistical Software (version 4.3.2; R Core Team 2023). The Shapiro–Wilk test confirmed the normality of the data in a majority of lipid measurements. Although a few datasets had a *p*-value < 0.05, the measurements of the same lipid species were normally distributed in other subjects, and *p* < 0.05 was not solely observed in one subject or lipid species. Therefore, we assumed that lipid measurements were normally distributed as a whole and decided to proceed with parametric analysis. Given the presence of heteroscedasticity (*p* < 0.05) as determined by Levene’s test, an analysis of variance (ANOVA) was performed across all conditions with Welch’s adjustment. A subsequent post hoc multiple comparison test using Dunnett’s method was conducted when ANOVA identified significant differences between samples. Differences were considered statistically significant at an adjusted *p*-value < 0.05.

## Results

### Mutational analysis of *SPTLC1* and identification of a* SPTLC1* variant in a patient with juvenile-onset ALS

The rare *SPTLC1* variants identified in this study are summarized in Table 1. No rare variants in *SPTLC1* were identified in patients with FALS. In patients with SALS, we identified two variants in *SPTLC1* as listed.

**Table 1:** The SPTLC1 variants identified in this study

Position (GRCh38/hg38)	Nucleotide change (NM_006415)	Amino acid change (NP_006406)	FALS (*n* = 40)	SALS (*n* = 413)	Control (*n* = 1163)	ToMMo 54KJPN AF	gnomAD Global AF
chr9:92112562	c.58G>A	p.Ala20Thr	0	1	0	ND	ND
chr9:92055467	c.718C>T	p.Arg240Cys	0	1	1	0.001934	0.000007
chr9:92038288	c.1214G>A	p.Arg405His	0	0	1	0.000092	0.000059
chr9:92034850	c.1288C>T	p.Arg430Cys	0	0	1	0.000157	0.000033

All the identified variants were heterozygous

*FALS* familial ALS, *SALS* sporadic ALS, *AF* allele frequency, *ND* not detected, *gnomAD* The Genome Aggregation Database v3 (https://nomad.broadinstitute.org/), *ToMMo* Tohoku Medical Megabank Organization, *jMorp* Japanese Multi Omics Reference Panel; https://jmorp.megabank.tohoku.ac.jp; 54KJPN

**Fig. 2:**
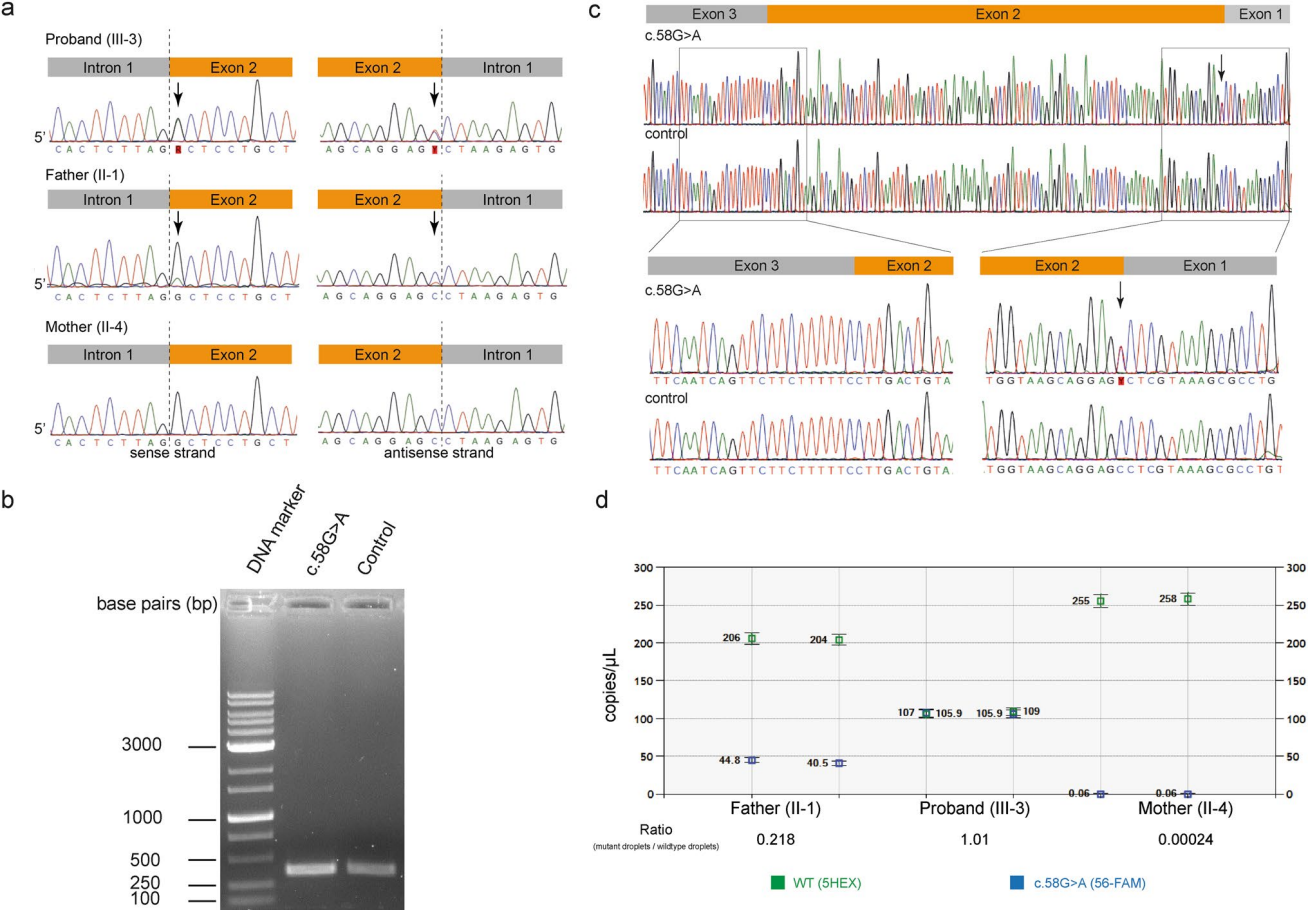
Genetic analysis of pedigree 1. **a** Direct nucleotide sequence analysis of the proband, father, and mother, in pedigree 1. A heterozygous variant (c.58G>A, p.Ala20Thr) in serine palmitoyltransferase long chain base subunit 1 (*SPTLC1*) was identified in the proband. The electropherogram showed a low-amplitude signal of c.58G>A in the father, suggesting a mosaic variant. **b** Reverse transcription-polymerase chain reaction (PCR) analysis of the c.58G>A variant. Electrophoresis of complementary DNA (cDNA) products. cDNA derived from the c.58G>A variant showed one band corresponding to that of the wildtype allele (374 bp). A band corresponding to exon 2 skipping (266 bp) was not observed. **c** Subsequent direct nucleotide sequence analysis of the cDNA product. The whole length of exon 2 was preserved in the c.58G>A variant. **d** Results of droplet digital PCR analysis. A duplicate assay was conducted for each sample, corresponding to the two dots. Mutant allele frequencies were calculated as 0.17, 0.50, and 0.00 in the father, proband, and mother, respectively

The first variant, c.58G>A, p.Ala20Thr (NM_006415.4), was identified as a heterozygous state in a 21-year-old Japanese female patient (Fig. 2a). This variant was not present in the population or in-house database of healthy individuals. Multiple in silico predictors indicated that the variant was deleterious (Supplemental Table 2). As the parents were asymptomatic, we initially suspected that this was a de novo variant.

The second variant, c.718C>T, p.Arg240Cys (NM_006415.4), was identified in a patient over the age of 40. This variant met the MAF criteria of < 0.01 in the population databases; however, it was also found in one of the in-house 1163 unrelated healthy individuals. This variant was classified as a variant of unknown significance in ClinVar (ClinVar ID 1026225). Based on curation using ClinVar, the affected status of the individual with the variant was unknown, with no data from functional studies, including lipid analysis. Therefore, we speculated that this variant was not a causative factor. The lack of detailed clinical data, family samples, and patient plasma samples precluded further investigation.

### Clinical features of the patient with c.58G>A, p.Ala20Thr *SPTLC1* variant

The patient with c.58G>A, p.Ala20Thr variant presented progressive muscle weakness beginning at the age of 6 years (Fig. 1a, pedigree 1, III-3). During elementary school, she had difficulty keeping up with her classmates during sports class. At the age of 13 years, scoliosis was noted during a medical checkup. She started playing the flute; however, owing to progressive dyspnea, she increasingly struggled to keep up with the entire band. At 18 years of age, she underwent surgery for prominent scoliosis. Electrophysiological examination at the previous institution revealed neurogenic changes in the forearms, suggesting a neurogenic etiology. Respiratory failure developed while she was still able to walk, requiring intermittent noninvasive positive pressure ventilation at the age of 18 years.

She visited our institution when she was aged 21 years. Physical examination showed low body weight (height, 153.0 cm; weight, 38.6 kg; body mass index, 16.5 kg/m^2^). A neurological examination revealed symmetrical weakness, dominant muscular atrophy of the distal upper extremities, and tongue atrophy. The Medical Research Council scale for muscle strength was grades 1–2 for the distal upper extremities and grade 4 for the neck, proximal upper extremities, and lower extremities. Atrophy was diffusely prominent in the intrinsic hand muscles without a clear split-hand sign. Tendon reflexes were absent in the upper extremities, with exaggerated Achilles tendon reflexes and positive Babinski sign on the left side. The Wechsler Adult Intelligence Scale-Third Edition (WAIS-III) showed a full-scale intelligence quotient (IQ) of 55, verbal IQ of 63, and a performance IQ of 53. Needle electromyography revealed acute and chronic denervation of the upper and lower extremities. Motor evoked potential studies showed prolonged central motor conduction time, supporting upper motor neuron dysfunction. Sensory nerve conduction was within the normal limits. Blood examination revealed mildly elevated creatinine kinase level (309 U/L), which was otherwise unremarkable. Cerebrospinal fluid examination results were unremarkable. Magnetic resonance imaging (MRI) of the brain revealed no abnormal-intensity signals in the precentral cortex or pyramidal tract on susceptibility-weighted imaging (Fig. 1c). MRI of the spinal cord did not reveal high-intensity signals or atrophy. The patient met the revised El Escorial criteria for probable ALS [13].

No other family members, including the proband’s parents, presented with neuromuscular symptoms. The parents underwent a full neurological examination, including cranial nerve examination, manual muscle test, and tendon and toe reflexes, and were confirmed to be intact. Consanguinity was not observed in the family. Despite a negative family history, the monogenic cause of ALS was compatible with early-onset disease in this patient.

### Absence of exon 2 skipping in the c.58G>A, p.Ala20Thr *SPTLC1* variant

The c.58G>T, p.Ala20Ser variant, a variant affecting the same nucleotide (Fig. 1b), has previously been reported to cause complete skipping of exon 2 [9]. In the electrophoresis, cDNA derived from the c.58G>A variant showed one band corresponding to that of the wildtype allele (374 bp). No band corresponding to exon 2 skipping (266 bp) was detected (Fig. 2b). Direct nucleotide sequence analysis of the cDNA product confirmed that the length of exon 2 was preserved (Fig. 2c). These results indicate that, unlike the c.58G>T variant in the same amino acid position, the identified c.58G>A variant does not cause exon 2 skipping.

### Identification of mosaicism in the asymptomatic father

The electropherogram of the direct nucleotide sequence showed a c.58G>A variant in the asymptomatic father with a low-amplitude signal indicative of a mosaic variant. Subsequent ddPCR analysis of genomic DNA from peripheral blood leukocytes revealed mutant allele frequencies of 50% in the proband, 17% in the father, and 0% in the mother, confirming mosaicism, as initially suggested by the electropherogram (Fig. 2d).

### Sphingolipid analysis in pedigree 1

**Fig. 3:**
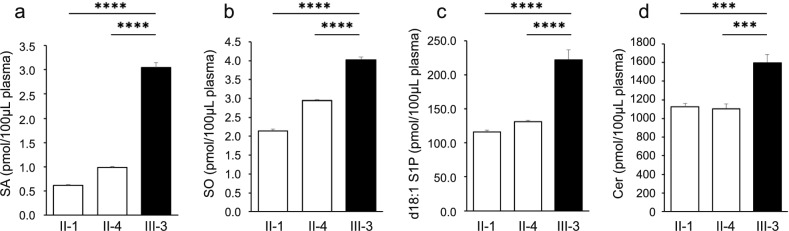
Results of the sphingolipid analysis. **a** Sphinganine (SA), **b** sphingosine (SO), **c** d18:1 sphingosine-1-phosphate (S1P), **d** total ceramides, including all lengths of fatty acid chains (Cer). SA, SO, d18:1 S1P, and Cer levels were significantly higher in the proband compared with the asymptomatic parents. d20:0 SA, d20:1 SO, deoxymethyl SO, and deoxy SO were not detected in any of the samples. ***p < 0.005, ****p < 0.0005

The results of sphingolipid analysis of the plasma samples from pedigree 1 are summarized in Fig. 3 and Supplemental Table 3. Investigation of long chain base form sphingolipids revealed significantly elevated levels of SA, sphingosine (SO), and SO-1-phosphate (S1P) (d18:1) in the plasma of the affected proband (III-3) compared with unaffected parents (II-1, II-4) (Fig. 3a–c). The levels of S1P (d20:1) and 1-deoxy-SA (deoxySA) were not significantly different among family members. 1-Deoxy-SO and 1-deoxymethyl-SO were not detected in any of the samples. Investigation of fatty acid-acetylated forms of sphingolipids revealed that the total ceramide (Cer) levels were significantly higher in the proband than in the asymptomatic parents (Fig. 3d). The levels of complex ceramides, including lactosylceramide, hexosylceramide, and sphingomyelin, did not significantly differ across family members. Deoxy-ceramide levels were not elevated in any of the samples. Notably, among the sphingolipids that were significantly elevated in the proband (SA, SO, S1P, and Cer), the father with mosaicism did not have consistently higher levels of these sphingolipids than the mother without mosaicism.

Furthermore, we found an alteration in the composition of ceramides in the affected patient. Ceramides are formed from fatty acid chains of various lengths that are bound to an SO base. The unaffected parents of the patient had a similar fatty acid chain length; however, the ratio of C24:1 ceramides increased in the affected individuals, together with a slight decrease in the ratio of C24:0, C26:0, and C26:1 ceramides (Table 2). The increase in C24:1 ceramides accounted for nearly half the increase in total ceramide levels in the proband.

**Table 2:** The levels of ceramide by fatty acid chain length and ratio to total ceramide levels (%)

	Levels of ceramide (pmol/100μL plasma)			Ratio to total ceramide levels (%)		
	II-1	II-4	III-3	II-1	II-4	III-3
C14:0	4.677 ± 0.157	5.100 ± 0.274	7.263 ± 0.730	0.42	0.46	0.45
C16:0	27.98 ± 0.98	32.41 ± 2.15	38.67 ± 1.71	2.49	2.93	2.42
C16:1	N.D. ± N.D.	N.D. ± N.D.	N.D. ± N.D.	N.D.	N.D.	N.D.
C18:0	59.09 ± 18.02	76.45 ± 5.84	90.71 ± 13.15	5.25	6.91	5.68
C18:1	14.79 ± 1.35	17.03 ± 1.08	23.14 ± 4.06	1.31	1.54	1.45
C20:0	13.13 ± 0.90	13.63 ± 2.54	19.13 ± 2.59	1.17	1.23	1.2
C20:1	1.745 ± 1.063	1.101 ± 0.390	2.024 ± 0.204	0.15	0.1	0.13
C22:0	233.6 ± 17.0	242.2 ± 51.3	322.7 ± 55.0	20.75	21.89	20.2
C22:1	2.675 ± 0.422	4.367 ± 0.528	5.530 ± 0.652	0.24	0.39	0.35
C23:0	108.5 ± 4.3	119.2 ± 10.2	148.8 ± 4.3	9.64	10.77	9.32
C23:1	N.D. ± N.D.	N.D. ± N.D.	N.D. ± N.D.	0.03	0.07	0.05
C24:0	322.7 ± 26.4	279.1 ± 20.3	371.9 ± 51.4	28.67	25.23	23.29
C24:1	206.3 ± 20.5	190.4 ± 11.2	408.9 ± 39.7	18.32	17.21	25.6
C26:0	5.788 ± 0.955	4.807 ± 1.347	3.864 ± 0.161	0.51	0.43	0.24
C26:1	124.8 ± 8.7	120.3 ± 12.9	153.5 ± 15.0	11.08	10.87	9.61
SUM	1125.9 ± 68.4	1106.2 ± 99.9	1597.0 ± 176.1	100	100	100

## Discussion

**Fig. 4:**
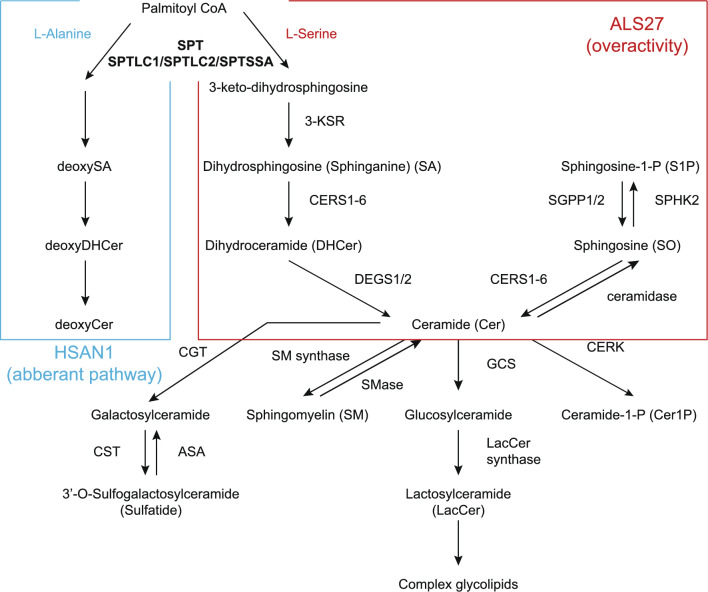
Metabolic pathway of sphingolipids. Normal serine palmitoyltransferase (SPT) uses l-serine and palmitoyl-CoA as substrates to form 3-keto-dihydrosphingosine. Abnormal SPT associated with HSAN1 uses l-alanine instead of l-serine as substrates, resulting in deoxysphinganine formation. In variants associated with amyotrophic lateral sclerosis (ALS), the abnormal SPT results in excessive production of sphynganines and ceramides, instead of deoxyphinganine formation. *ASA* arylsulfatase A, *CERK* ceramide kinase, *CERS* ceramide synthase, *CGT* ceramide galactosyltransferase, *CST* cerebroside sulfotransferase, *DEGS* dehydroceramide desaturase, *GCS* glucosylceramide synthase, *3-KSR* 3-ketosphinganine reductase, *SGPP* sphingosine 1-phosphate phosphatase, *SPT* serine palmitoyltransferase

Here, we demonstrated the pathogenicity of the c.58G>A, p.Ala20Thr *SPTLC1* variant based on sphingolipid analysis. *SPTLC1* encodes a subunit of SPT, an enzyme that catalyzes the first and rate-limiting process in sphingolipid synthesis (Fig. 4) [21, 22]. A physical map of SPTLC1 and associated pathogenic variants are outlined in Fig. 1b [9, 10, 23]. Pathogenic variants of *SPTLC1* are associated with HSAN1 [8]. Normal SPT uses l-serine and palmitoyl-CoA as substrates to form 3-keto-dihydrosphingosine. The abnormal SPT associated with HSAN1 uses l-alanine instead of l-serine as a substrate, resulting in deoxySA formation. ALS-associated variants in *SPTLC1*, clustered in exon 2, which encodes the first transmembrane domain of SPTLC1, result in excessive production of SA and ceramides, instead of deoxySA formation [9, 10]. The c.58G>A, p.Ala20Thr variant has been reported in one patient with sporadic juvenile ALS [24]; however, its biochemical consequences and effects on splicing have not been evaluated in that patient. In this study, we confirmed that SA, SO, and Cer levels were elevated in the affected patient. The clinical features were also in line with those of previously reported cases; however, the interpretation of WAIS-III requires further discussion. Cognitive decline is an infrequent feature of *SPTLC1*-associated ALS, which has been described in only one previously reported case [10]. Specifically, the previously reported patient with the same c.58G>A variant did not present with cognitive decline. Although the WAIS-III revealed a low IQ, this finding was disproportionate to her intellectual performance in daily life, such as passing a university entrance examination. Therefore, we are reluctant to conclude that this patient had cognitive decline. Fatigue was observed in the patient during the long examination period, which may have affected the scores.

Notably, we found an alteration in the composition of ceramides in the affected patient. The unaffected parents of the patient had a similar fatty acid chain length; however, the ratio of C24:1 ceramide increased in the affected individual. This implies that an abnormal increase in ceramides may occur in different proportions to the physiological composition. Previous studies on ceramide levels in *SPTLC1*-associated juvenile ALS have not specifically investigated C24:1 levels. Further analyses of sphingolipids in patients with *SPTLC1*-associated ALS are necessary to elucidate the effects of *SPTLC1* variants on ceramide synthesis.

The c.58G>T p.Ala20Ser variant is a previously reported pathogenic variant of *SPTLC1*. cDNA derived from cultured skin fibroblasts or whole blood derived from the patient with the c.58G>T variant resulted in two bands, corresponding to a full-length transcript and an internally deleted transcript, neither of which contained the missense variant p.Ala20Ser, suggesting complete exon 2 skipping [9]. The c.58G>A, p.Ala20Thr variant affecting the same nucleotide had different consequences on splicing. The results of multiple in silico splice effect prediction tools, including BDGP/Splice Site Prediction by Neural Network [25], Splice AI [26], MaxEnt Scan [27], and Alternative Splice Site Predictor [28], indicated a higher likelihood of a splice defect in the c.58G>T variant compared with the c.58G>A variant, consistent with the results of cDNA analysis (Supplemental Table 4). Notably, Ala20 is a site that makes contact with the third membrane-spanning domain of ORMDL [9, 29, 30]. Therefore, a single amino acid substitution at this site may be sufficient to cause reduced interaction with ORMDL, leading to the loss of ORMDL-mediated inhibition of SPT activity. We should mention that cDNA analyses in our study and the previous study [9] were conducted with non-neural tissue, and may not reflect the actual splicing of transcripts in neural tissue.

Finally, we found that the father with mosaic variant of *SPTLC1* may be asymptomatic. Mosaic variants are acquired during postzygotic cell division, resulting in different genotypic variants among cells derived from the same zygote. In some diseases, mosaic variants may lead to overt clinical presentation, similar to hereditary cancer syndromes [31], cutaneous diseases [32], and muscular dystrophy [33], but mosaic variants may also be found in clinically asymptomatic individuals [34]. In this study, the asymptomatic father had a relative abundance of 17% mutant *SPTLC1* alleles in the peripheral blood cells. A previous study also included an asymptomatic parent with mutant *SPTLC1* in one of 49 next-generation sequence reads from saliva-derived DNA [10], further supporting the absence of symptoms in individuals with mosaicism. One possible explanation for the absence of symptoms in individuals with mosaic variants is that the abundance of mutant alleles is low in the central and peripheral nervous systems, which are the main systems affected by the disease. Another possibility is that the relative abundance of abnormal SPTLC1 is insufficient to cause ALS. Contrary to our expectations, the mosaicism in the father did not lead to mildly elevated sphingolipid or ceramide levels. This suggests that 17% abundance of *SPTLC1* mutant allele does not cause biochemical consequences in lipid metabolism or associated neurotoxicity. Furthermore, this finding may have important implications for treatment development; even a mild reduction in mutant SPTLC1 may have a drastic effect on the phenotype. Further investigations are necessary to determine whether there is a threshold for the relative abundance of mutant alleles in the clinical presentation of ALS.

## Conclusion

We identified a pathogenic c.58G>A, p.Ala20Thr *SPTLC1* variant in a patient with juvenile ALS. The lipid analysis results are consistent with those of previous studies on *SPTLC1*-associated ALS. Together with the recent identification of *SPTLC2*, our findings support the role of SPT and sphingolipid metabolism in the pathogenesis of ALS. *SPTLC1*-associated juvenile ALS may have inherited a pathogenic variant from an asymptomatic parent with mosaicism. Further studies are necessary to determine the clinical effect of mosaic variants of *SPTLC1*.

## Supplementary Information

Below is the link to the electronic supplementary material.

Supplementary file1 (PDF 161 kb)

## Data Availability

Data supporting the findings of this study are available upon request from the corresponding author.
