# Supplementary material for: Genetic and functional analyses of SPTLC1 in juvenile amyotrophic lateral sclerosis
Source: J Neurol. 2024 Dec 12;272(1):36. doi: 10.1007/s00415-024-12776-5 (PMC11638311; doi:10.1007/s00415-024-12776-5)
Supplement: Supplementary file 1 — Supplementary file1 (PDF 161 kb) [file 415_2024_12776_MOESM1_ESM.pdf]

Supplemental Methods.

Genetic analysis

Prior to this study, all patients had been screened for variants or expansions of known ALS-associated genes.[1, 2] The remaining 43 families with FALS and 445 patients with SALS not carrying causative variants were enrolled in this study. Whole-genome sequence was performed for the proband (pedigree1, III-3) using Illumina TruSeq DNA PCR-free library kits resulting in paired-end 150 bp reads on NovaSeq6000 sequencer (Macrogen). Whole-exome sequence analysis was performed according to a previously established workflow.[1] No repeat expansion mutation in *C9ORF72* was detected by repeat-primed PCR analysis in these patients.

Sphingolipid measurements.

The internal standard materials, purchased from Avanti Polar Lipids (Alabaster, AL, USA) for sphingolipid analysis, comprised the internal standard mixture (ceramide/sphingoid internal standard mixture I), D-erythro-sphinganine-d7, D-erythrosphingosine-d7, D-erythro-sphingosine-d7-1-phosphate, 1-deoxy-sphinganine-d3 (m18:0), 1-deoxymethyl-sphinganine-d5 (m17:0), N-lauroyl-dihydroceramide (C12DHCer), N-C12–1-deoxy-dihydroceramide (C12doxDHCer), N-C12–1-deoxyceramide (C12doxCer), N-C12-1-deoxymethyl-dihydroceramide (C12doxmeDHCer), and N-C12-1-deoxymethyl-ceramide (C12doxmeCer). For the lipid extraction and analysis, 300 µl of cold water was mixed with 100 µl of each plasma sample. After adding 1.5 ml of chloroform/methanol (1:2, v/v), the internal standard mixture was added to the homogenate and incubated at 48 °C overnight. After adding 150 µl of 1 M potassium hydroxide in methanol, the mixture was incubated for 2 h at 37 °C, and then neutralized by adding glacial acetic acid. For the analysis of base-form sphingolipids (sphingoid base: sphinganine [SA], sphingosine [SO], sphingosine-1phosphate [S1P], 1-

deoxy-sphinganine [deoxy SA], 1-deoxy-sphingosine [deoxy SO], 1deoxymethyl-sphinganine [deoxymethyl SA], and 1-deoxymethyl-sphingosine [deoxymethyl SO]), samples were centrifuged at 1,500 × g for 10 min to obtain the supernatant. The residue was re-extracted with 1 ml of chloroform/methanol (2:1, v/v) and centrifuged at 1,500 × g for 10 min. Both supernatants were then combined for further analysis. For the analysis of fatty-acid-acylated-form sphingolipids (dihydroceramide [DHCer], ceramide [Cer], 1-deoxy-dihydroceramide [deoxy DHCer], 1-deoxy-ceramide [deoxy Cer], 1-deoxymethyl-dihydroceramide [deoxymethyl DHCer], 1-deoxymethyl-ceramide [deoxymethyl Cer], lactosylceramide [LacCer], hexosylceramide [HexCer] and sphingomyelin [SM]), 1 ml of chloroform and 2 ml of water were added to the samples; the samples were vortexed well and centrifuged at 1,500 × g rpm for 10 min to obtain a two-phase system, the aqueous top phase and the organic bottom phase, from which the lipids were extracted. The top phase was re-extracted with 1 ml of chloroform and centrifuged at 1,500 × g for 10 min, after which the organic phases were combined. These lipid extracts were dried under an N2 stream and stored at -20 °C until further use. The dried residue was dissolved in the mobile phase solvent immediately before LC-ESIMS/MS analysis using the Nexera X2 HPLC system (Shimadzu, Kyoto, Japan) and QTRAP 4500 (AB SCIEX, Framingham, MA, USA).

1. Naruse H, Ishiura H, Mitsui J, et al. Burden of rare variants in causative genes for amyotrophic lateral sclerosis (ALS) accelerates age at onset of ALS. J Neurol Neurosurg Psychiatry. 2019;90:537–542.

2. Naruse H, Ishiura H, Mitsui J, et al. Molecular epidemiological study of familial amyotrophic lateral sclerosis in Japanese population by whole-exome sequencing and identification of novel HNRNPA1 mutation. Neurobiology of Aging. 2018;61:255.e9-255.e16.

Supplemental Table 1. Primers and probes for the analysis using direct nucleotide sequence, cDNA and droplet digital PCR.

Primer/probe name	Sequences	Primer/Probe length
**Direct nucleotide sequence analysis**		
SPTLC1_ex2_F	5'-GAGCCACCACAAATCCTTTC	20
SPTLC1_ex2_R	5'-GTGAGGGAGAAATTGCCTGC	20
**cDNA analysis**		
SPTLC1_cDNA_F	5'-AGTGGGTTCTGGTGGAGATG	20
SPTLC1_cDNA_R	5'-CCCCACGCCATACTTCTTTAG	21
**ddPCR analysis**		
SPTLC1_ddPCR_F	5'-TCCAGAGGATCAGAATCC	18
SPTLC1_ddPCR_R	5'-GGTGTTAGAAGTGTATGTTTC	21
wtSPTLC1_probe	5'-(5HEX) ACTCTTAGGCTCCTGCT	17
mSPTLC1_probe	5'-(56-FAM) CACTCTTAGACTCCTGCT	18

cDNA, complementary DNA; ddPCR, droplet digital PCR.

Supplemental Table 2. In-silico prediction of damage of this variant.

Analysis	Score	Prediction
CADD	26.9 (PHRED)	Damaging
M-CAP	0.067	Possibly pathogenic
FATHMM	-3.51	Damaging

CADD, Combined Annotation Dependent Depletion; FATHMM, Functional, Molecular and Phenotypic Consequences of Amino Acid Substitutions Using Hiddent Markov Models; M-CAP, Mendelian Clinically Applicable Pathogenicity.

Supplemental Table 3. Lipid analysis of the plasma samples from pedigree 1.

Base form sphingolipids (pmol/100µL plasma)

	d18:0 SA			d18:1 SO			d18:1 S1P			d20:1 S1P			deoxy SA			deoxymethyl SA		
II-1	0.616	±	0.034	2.146	±	0.095	116.3	±	5.52	1.137	±	0.070	0.043	±	0.003	0.558	±	0.089
II-4	0.984	±	0.047	2.941	±	0.047	131.4	±	4.00	1.055	±	0.079	0.077	±	0.012	0.157	±	0.024
III-3	3.043	±	0.207	4.023	±	0.162	222.4	±	28.94	1.288	±	0.104	0.065	±	0.005	0.140	±	0.013

Fatty-acid acetylated forms (pmol/100µL plasma)

	Cer			C24:1 Cer			DHCer			LacCer			HexCer			SM		
II-1	1125.9	±	68.4	206.3	±	20.5	140.7	±	17.7	356.5	±	11.7	1431.6	±	223.7	47649.9	±	11462.4
II-4	1106.2	±	99.9	190.4	±	11.2	256.7	±	20.0	299.8	±	31.2	1200.9	±	239.5	30459.5	±	900.2
III-3	1597.0	±	176.1	408.9	±	39.7	267.9	±	14.7	342.8	±	27.9	1748.0	±	105.0	34931.9	±	4533.1

Deoxysphingolipids, fatty-acid acetylated forms (pmol/100µL plasma)

	deoxy Cer			deoxymethyl Cer			deoxy DHCer			deoxymethyl DHCer		
II-1	3.108	±	0.108	1.905	±	0.092	9.662	±	0.507	0.208	±	0.012
II-4	3.126	±	0.164	1.763	±	0.127	16.779	±	0.310	0.274	±	0.013
III-3	1.600	±	0.009	2.242	±	0.144	9.114	±	0.356	0.461	±	0.011

* d20:0SA, d20:1SO, deoxymethylSO, deoxySO were not detected in any of the samples.

SA, sphinganine; SO, sphingosine; S1P, sphingosine-1-phosphate; deoxy SA, 1-deoxy-sphinganine; deoxymethyl SA, 1-deoxymethyl-sphinganine; Cer, ceramide; DHCer, dihydroceramide; LacCer, lactosylceramide; HexCer, hexosylceramide; SM, sphingomyelin; deoxy Cer, 1-deoxy-ceramide; deoxymethyl Cer, 1-deoxymethyl-ceramide; deoxy DHCer, 1-deoxy-dihydroceramide; deoxymethyl DHCer, 1-deoxymethyl-dihydroceramide.

Supplemental Table 4. In-silico prediction of the effect on splicing.

**Analysis**	**WT**	**c.58G>A**	**c.58G>T**
NNSplice	0.92	**0.86**	0.71
Splice AI (acceptor loss)	-	**0.01**	0.15
MaxEnt Scan	6.9	**6.14**	5.32
Alternative Splice Site Predictor	5.816	**4.765**	3.599
